# Evaluating feasibility of using national registries for identification, invitation, and ultrasound examination of persons with hereditary risk for aneurysm disease—detecting abdominal aortic aneurysms in first degree relatives (adult offspring) to AAA patients (DAAAD)

**DOI:** 10.1186/s40814-022-01196-9

**Published:** 2022-12-12

**Authors:** Rebecka Hultgren, Nina Fattahi, Olga Nilsson, Sverker Svensjö, Joy Roy, Anneli Linne

**Affiliations:** 1 Department of Vascular Surgery, Karolinska University Hospital Stockholm, Stockholm, Sweden; 2 Stockholm Aneurysm Research group, STAR, Department of Molecular Medicine and Surgery, Karolinska Institutet, Stockholm, Sweden; 3 Department of Clinical Science and Education, Karolinska Institutet at Södersjukhuset, Stockholm, Sweden; 4 Section of Vascular Surgery, Department of Surgery, Södersjukhuset, Stockholm, Sweden; 5 Department of Surgical Sciences, Uppsala University, Uppsala, Sweden; 6 Centre for Clinical Research, Falun, Sweden

**Keywords:** AAA, Heredity, National registry, Multi-generation registry, Screening, Mortality, Sex differences

## Abstract

**Background:**

Sweden and the UK invite all 65-year-old men to a population-based ultrasound-based screening program to detect abdominal aortic aneurysms (AAA). First-degree relatives of patients with AAA are reported to have an increased risk to develop AAA, both women and men, but are not invited to screening. The “Detecting AAA in First Degree Relatives to AAA patients” (DAAAD) was designed to detect the true prevalence in adult offspring to AAA patients and to evaluate if national registries could be used for identification of index persons and their adult children with a high risk for the disease. The aim of this study is to summarize the design and methodology for this registry-based study.

**Methods:**

The study is based on a registry-based extraction and identification of a risk group in the population with a subsequent identification of their adult offspring. The targeted risk group suffers a heredity for a potentially lethal disease, AAA (*n* = 750) and matched control group without heredity for AAA is also identified and invited (*n* = 750). The participation rate in the population-based AAA screening program for men is 75% regionally. This population is younger and have a lower prevalence. A participation rate of 65% is considered clinically adequate. For the DAAAD study, a stratified analysis of the primary outcome, prevalence, will be performed for women and men separately. Two other planned projects are based on the material: firstly, evaluation of the anxiety for disease and health-related quality of life (HRQoL) and, secondly, the cost-effectiveness of the study.

**Discussion:**

In conclusion, this feasibility study will be instrumental in supporting the development of a possible new model to invite persons with high risk to develop hereditary rare diseases. To our knowledge, this is a unique, safe, and most likely to be a cost-efficient model to invite targeted risk groups for selected screening. If the study design and the results are shown to be cost-effective at the detected participation rate and prevalence, it should be further evaluated and adopted to a national screening program. The model also invites both women and men, which is unique for this specific patient group, considering that all population-based screening programs only include men.

**Trial registration:**

This trial is registered at the website of Clinical Trials. ClinicalTrials.gov identifier, NCT4623268

**Supplementary Information:**

The online version contains supplementary material available at 10.1186/s40814-022-01196-9.

## Introduction

**Fig. 1:**
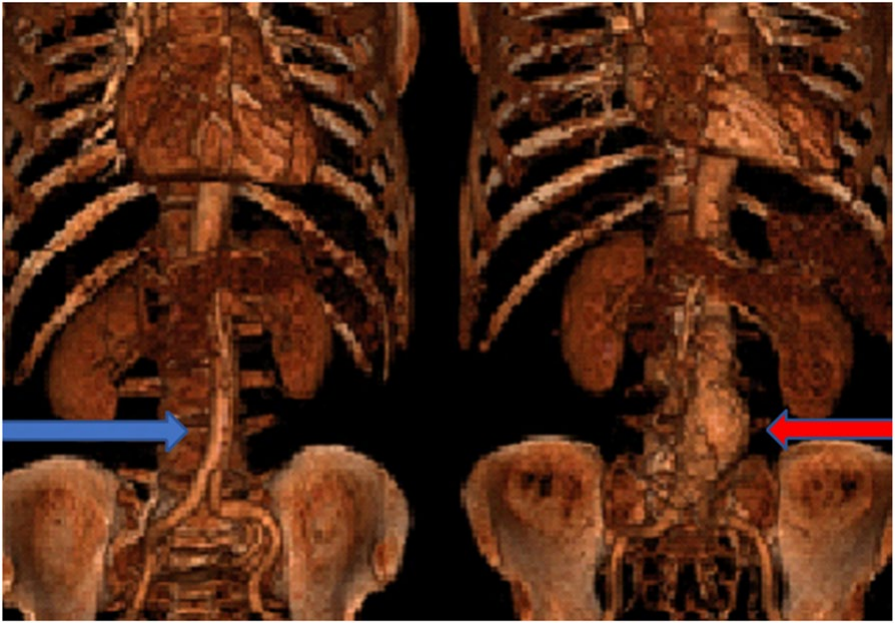
Radiological image of a two persons, one normal aorta (blue arrow) and an abdominal aortic aneurysm (red arrow) in the abdomen. It shows the local potentially lethal but asymptomatic bulge of the aorta below the renal arteries. If detected by systematic screening before it ruptures, it can be treated with a vascular intervention

Abdominal aortic aneurysm (AAA) is an asymptomatic widening of the infrarenal aortic diameter to ≥ 3 cm [1]. Male sex, increasing age, smoking, hyperlipidemia, cardiovascular diseases, aneurysmatic disease, and heredity are risk factors commonly detected in cohorts of patients with AAA [2–4]. This multifactorial disease has life-threatening consequences; if rupture occurs, mortality is 100% if left untreated (Fig. 1). The risk for rupture is closely related to the size of the AAA and identification of patients with smaller AAA is therefore crucial. The proportion of women among patients presenting with an intact aneurysm is low compared to men (1:4-6 ratio). The proportion of women among patients presenting with a rupture is considerably larger (1:3); women also have a higher complication rate, higher turn-down rate for surgery, and more challenging aneurysm anatomy [5, 6]. Since 2016, the Swedish National Board of Health and Welfare (NBHW) recommend population-based screening of 65-year old men, based on a reported 40% reduction in aneurysm-related death in screened men within 7 years [7]. The incremental cost-efficiency ratio was 8000 Euro/QALY [8].

While current successful national screening programs are aimed at men above 65, they do not account risk groups among women or siblings, such as the UK national AAA screening program [9]. Even if AAA is a multifactorial disease, most genetic studies have failed to identify one true pathway [1]. A Swedish and Danish twin study found a heritability of 70% of the total variance of liability for AAA. The remaining variance was explained by nonshared environmental factors [3, 10].

The reported extremely high prevalence rates in siblings should be compared to the prevalence of AAA in the general population: 6% in sisters vs 0.5% in women and 17% in brothers vs. 2% in men within the same age groups [11, 12]. It is not known if this is similar for adult offspring, but basic knowledge of hereditary traits in other disease groups suggests that it would be [13, 14]. Although the prevalence is higher and the cost-efficiency of screening siblings now is estimated to be at least similar to screening of 65-year-old men, no systematic attempt to screen or detect AAA is performed for this high-risk group [15].

Also, no systematic targeted screening of first-degree relatives (FDR) to AAA patients is presently performed although recommended in international guidelines [4, 16]. In a local FDR screening program, less than 10% of siblings had been screened before the invitation [11]. It is highly probable that the adult offspring of AAA patients have an even lower awareness due to younger age at the index person onset of disease, especially since such screening must be performed later in life (above 45–50 years of age). It has not been evaluated if this risk group has anxiety associated with the knowledge of their risk for a lethal disease. Most importantly detection of the hereditary trait will also address and specifically includes women at risk that are otherwise never invited for screening of AAA.

The possible negative effects of screening on health-related quality of life (HRQoL) in elderly men has been analyzed, showing quite diverging results [17, 18]. It is important that this information is collected prior to the diagnosis at screening in order to understand the wellbeing before the AAA diagnosis and the potential influence of anxiety for a future lethal disease after the diagnosis. The overarching aim with the project is to explore the hereditary aspects of AAA in adult offspring with a different methodology. This summary aims to describe the design, use, and feasibility of an identification, invitation, and subsequent ultrasound investigation of adult offspring at high risk for disease based on information collected from national registries. If this design and structure is feasible and participation is satisfactory, it will facilitate future design of population-based screening in order to decrease the high risk of aneurysm rupture and death that this high-risk group suffers today.

## Material and methods

### Study design and setting

The study design is a cross-sectional point prevalence study.

**Fig. 2:**
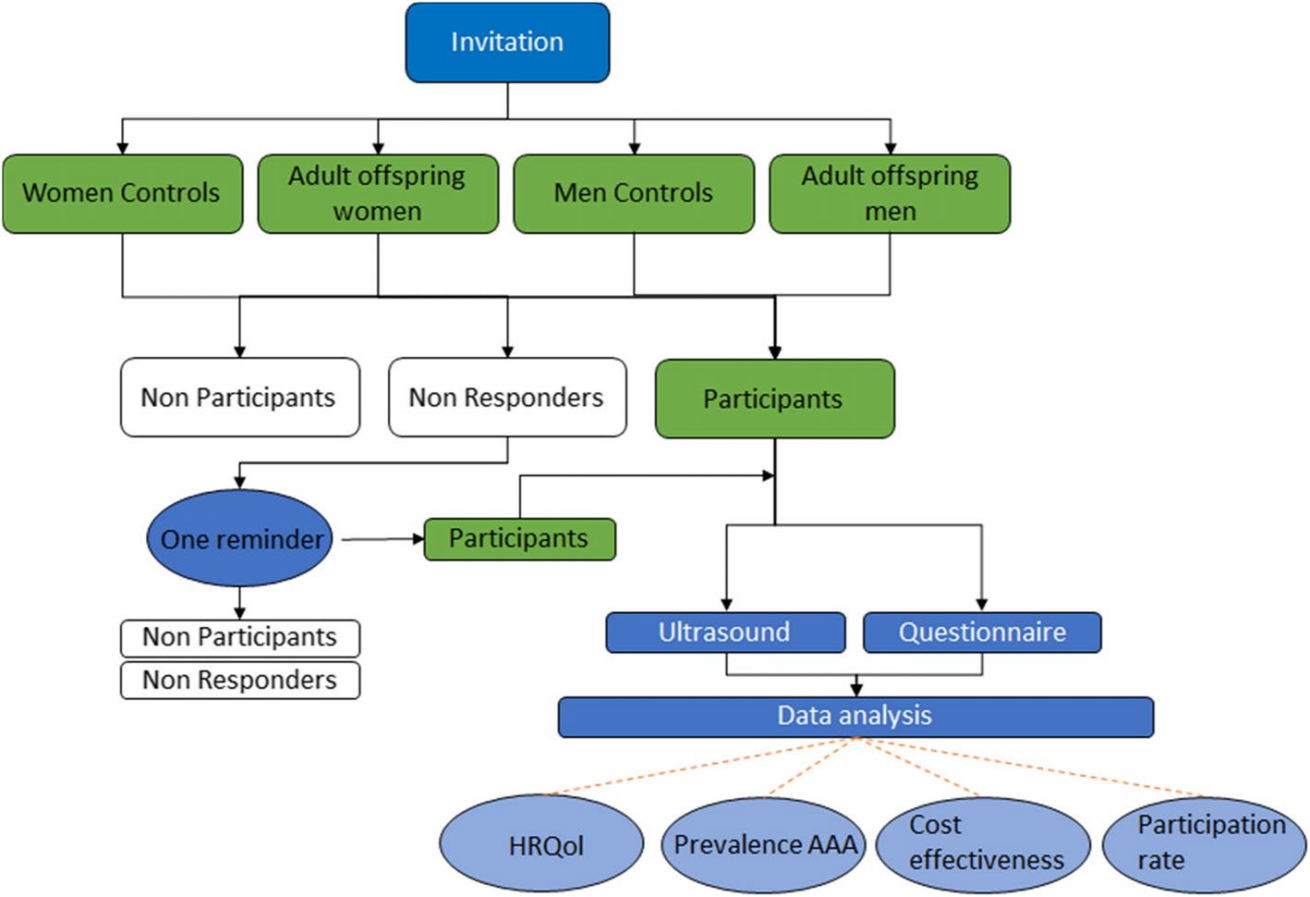
Flowchart of the DAAAD project and the four endpoints

The first objective was to evaluate if it is feasible to identify and invite persons in the population to participate in a targeted screening program by finding risk groups in national registries. Participation rate is considered as a factor that mirrors acceptance in the invited population for the screening program. The primary aim of the actual DAAAD study is to investigate the prevalence of AAA in adult offspring to AAA patients compared to matched controls for women and men. The second aim is to investigate the awareness of and anxiety for the hereditability for AAA in adult offspring to AAA patients compared to controls. The third aim is to evaluate the cost-efficiency of the program, compared to sibling screening and screening in 65-year-old men (Fig. 2).

The outcome measures are as follows: (1) feasibility of study design is measured by achieving a target population with an expected participation of 65% with a 95% confidence interval (63,67); (2) point prevalence of AAA in a risk group of adult offspring to AAA patients as compared to a matched control group; (3) patient-reported outcomes in risk groups based on the following questionnaires: Hospital Anxiety and Depression Score (HADS), EQ-5D, and a study-specific questionnaire on heredity, including awareness of their risk for AAA; and (4) cost-effectiveness of such a national program based on prevalence and EQ-5D.

### The detection route

#### The Swedish registries

Three registries have been used to extract the cohort of adult off-spring and the control group: (1) the National Patient register (NPR) which includes the Swedish Inpatient and Outpatient registry data and the National Hospital Discharge registry, (2) Statistics Sweden, and (3) the multigeneration register. The NPR at the NBHW started to collect data in 1964, has full coverage since 1987, and since 2001 also includes outpatient care. Furthermore, it includes all persons treated in hospitals in Sweden, with 100% coverage, and is based on the individual personal identification number (PIN) and on the International Classification of Diseases (ICD). No patients are treated for AAA outside the public health care system in Sweden, since these are listed in this register for reimbursement reasons. The register Statistics Sweden includes all persons living in Sweden since 1968. The multi-generation register is held by Statistics Sweden, which includes all persons living in Sweden and residents born since 1931. These are linked to parents, children, and siblings.

#### Identification of the index person

The identification of the AAA patients, i.e., the index persons (born 1900–1953) in the NBHW registry generated 69,000 persons with the relevant ICD-codes identified in September 2020 (Supplemental Table). These persons had 137,000 registered children, 116,000 of the women were born 1939–1969, and the men 1939–1974. Of these, 18,131 were registered inhabitants in County Stockholm as of 31 December 2019. The control population was selected as adult offspring to the matched non-AAA index persons (born 1900–1953) from Statistics Sweden and thereafter matched for every adult offspring: 1 person matched based on age, sex, and region.

**Fig. 3:**
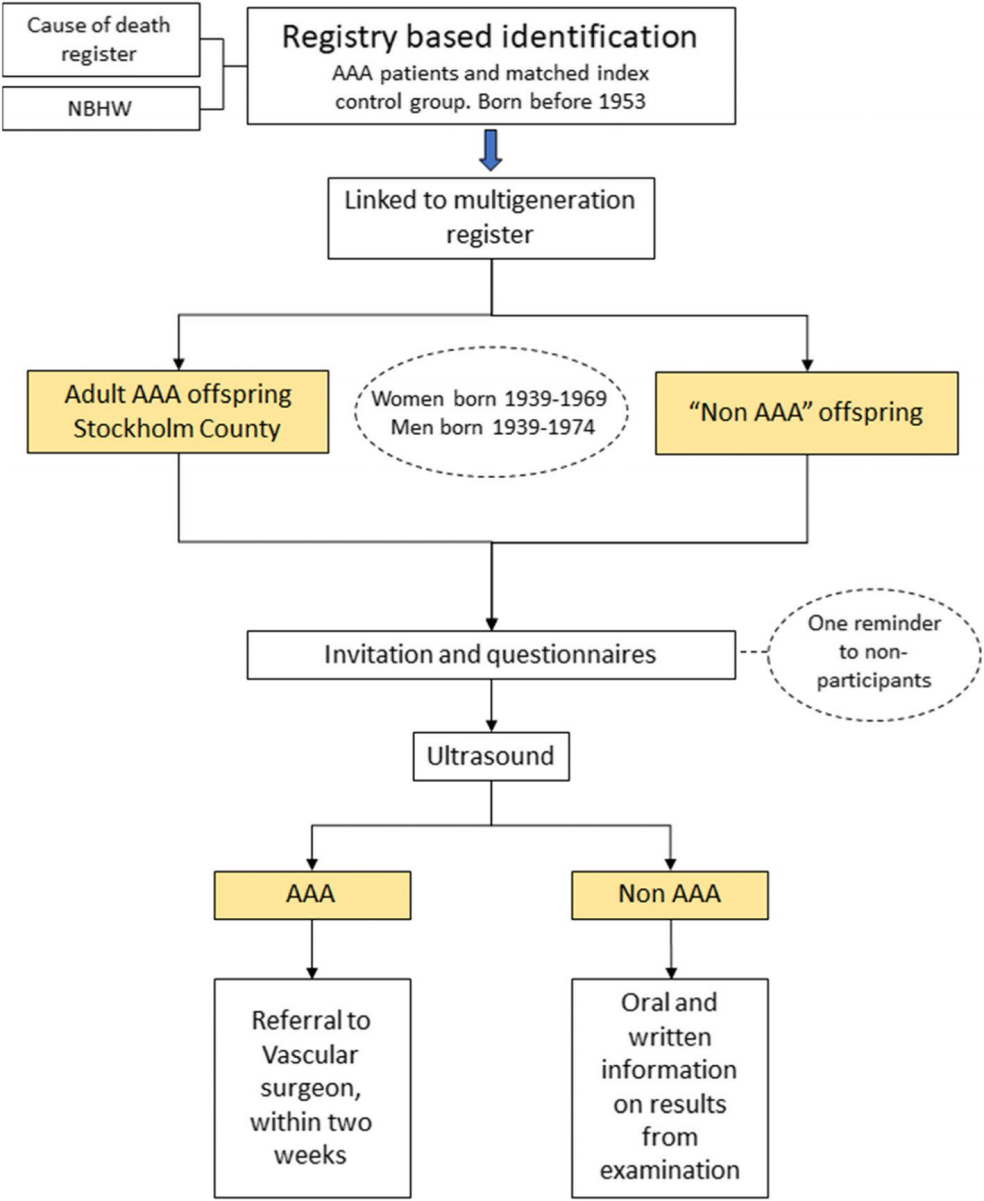
Flow chart of identification route of index person (patient with AAA) and adult offspring to index person

From this cohort of 18,131 persons, a random selection of 3800 persons was selected: 2000 women and 1800 men. The selected mean age for the extraction was 65 years for both women and men with an age distribution, mirroring the population. The contact details to send invitations was thereafter submitted to the core center at Karolinska (Fig. 3).

#### Letter and invitation

The invitation does not contain information on the persons’ specific risk or index person. The matched controls are invited by an identical letter, thereby decreasing the risk of bias. Basic information on the intention of the project is enclosed along with a pre-scheduled invitation to an abdominal ultrasound. To non-participants, one reminder is sent after 2 months. The participants can contact the core center on a specific telephone number to reschedule their appointment or ask questions. The invitation also includes the PI’s e-mail address. Successful participation is defined as invited persons who signed the consent form and filled in the questionnaires as well as being examined by ultrasound.

#### The study procedure

The Stockholm County has been running an efficient validated population-based screening program for 65-year-old men run since 2010. The scheduled screening process in DAAAD replicates the process within the national screening program. Participants in the study are investigated with the same machines and by the same investigators as men in the national population-based screening program for 65-year-old men. The ultrasound examinations are performed at two centers.

All invited persons will receive an invitation for a free ultrasound examination of their aorta and a telephone contact number. Since DAAAD is a scientific evaluation, a core facility located at the university hospital was formed with a dedicated research nurse for the project. This invitation also included information on the trial, a consent form, a pre-booked ultrasound appointment, and a personal code to access the web-based questionnaires. The code was provided along with a short instruction. Three questionnaires are included: a questionnaire regarding their general health, awareness, and anxiety regarding the risk for AAA disease, a second questionnaire with HADS [19], and finally the EQ 5D [20, 21].

At the ultrasound screening, the persons are identified by a research nurse, informed consent form is collected, and the questionnaire fulfillment is made before the aortic ultrasound is made. One can fill in the web-based forms prior to attending the ultrasound unit or do it at the screening unit, in paper form or on a tablet with the personal code. Thereafter, a quick pain-free ultrasound examination of the aortic diameter is performed, usually completed within 2–5 min. The majority will obtain oral and written information that the aorta is normal. The persons with an aortic diameter exceeding 30 mm are categorized as “patients with AAA” and will automatically be referred into the general care flow for diagnosed AAA patients at the two vascular regional units, parallel to the care flow of screened men.

All collected information from all participating persons is transferred to a computerized locked web system for this project. All persons are only registered according to an anonymous study ID, and all data is anonymized according to standard routines for person-registers in health care and research facilities. The databank will include information on the following: age and sex of index person. Summarized collection of data from all participants included the following: age, sex, self-reported comorbidity, general health measurements (weight, length), QoL information obtained from questionnaires, and aortic diameter in mm.

#### Sample size

The prevalence and onset of disease is different in women and men, and the sample size is evaluated separately. For men with an alpha of 0.05, power 0.80, an estimated prevalence of 7% in offspring, and 1.0% in controls, a required minimum of 166 persons in each group is requested, but 65% participation implies 350 invited men in each group. For women with an alpha of 0.05, power 0.80, an estimated prevalence 5% in offspring, and 0.5% in controls, a required minimum of 206 persons in each group is requested, estimating 400 women in each group. The participation rate is expected to be approximately 65%, based on 1500 examined individuals; with a 95% confidence interval between 63 and 67% similarly, which is lower than the population-based screening (75%). A lower level of expected participation is chosen due to the reported lower participation rate in other screening programs with younger individuals.

#### Preliminary plan for analysis

All data will be deidentified and transferred to an SPSS and STATA file for statistical analysis. In the basic analysis on prevalence, women and men will be presented separately. Prevalence is estimated on persons obtaining the AAA diagnosis at the screening for DAAAD, but the prevalence of AAA in persons prior to DAAAD will also be presented. The same applies to missing cases and non-participants. Per-protocol analysis will be performed, based on the four groups: sex (women/men) and heredity (yes/no). For comparisons of independent groups, Student’s *t* test will be used for normally distributed data and Mann-Whitney for non-parametric data. Categorical data will be analyzed by Fischer’s test where possible. Continuous variables are presented as means (SD) while categorical variables are presented as counts and proportions as appropriate. All continuous variables will be summarized using the following descriptive statistics: *n* (non-missing sample size) and mean and standard deviation. The frequency and percentages (based on the non-missing sample size) of observed levels will be reported for all categorical measures. In general, all data will be listed, sorted by groups and stratified by sex. All summary tables will be structured with a column for each group (adult offspring or control, women and men). The primary endpoint is as follows: prevalence will be presented for each strata and group. Derived variables to be included in the analyses are the subscales of the HADS instrument, HADS-Anxiety (HADS-A), and HADS-Depression (HADS-D) as well as the subscales of the EQ-5D. *P*-values ≥ 0.001 will be reported to 3 decimal places; *P*-values less than 0.001 will be reported as “< 0.001.” All statistical analyses will be performed in SPSS version 27 and in Stata (Version IC.16.1 Stata Corp, College Station, TX) when performed in collaboration with the reviewing statistician (Sverker Svensjö, Uppsala). The reviewing statistician will have an overview of the entire analyses. The cost-efficiency analysis will demand specific expertise and will be validated against other screening programs using a Markov Model. The model, analyzed by micro-simulation, simulates real-life screening, based on estimated, as well as published risks and costs. It aims to determine the incremental cost-efficiency ratio (ICER) in Euros/QALY for screening of targeted FDRs. Women and men will be analyzed separately. The model-based analysis will compare the strategy of screening targeted FDRs versus standard care (not screening individuals at risk). Outcome, in addition to ICER, will be absolute and relative risk reduction for AAA death, as well as quantifying the number of AAA events associated with each strategy. The model is derived from a previously validated and published AAA screening model, modified to suit the context of assessing FDR screening. The model was developed in the TreeAge Pro 2017 Healthcare software package (TreeAge Software, Williamstown, MA, USA) [15].

#### Trial progression and invitation of persons

The study was evaluated and approved by the Swedish Ethical Review Authority in April 2019. The study was then registered at the Clinical Trials website (ClinicalTrials.gov identifier, NCT4623268) (Table 1, Figs. 2 and 3). The process of the registry-based extraction started formally with the NBHW in August 2019. The internal process with approval of application was finalized in May 2020, and the extraction from the merged files was made at the NBHW. The completed file of identified and adult off-spring and matched controls was submitted in September 2020.

**Table 1:** Study assessments included in the web-based study protocol

Visit	Baseline
Time window	Before examination
Informed consent	Before examination
Demographic data	Before examination
Medical history	Before examination
Concomitant medication	Before examination
Questionnaires	Before examination
US aortic diameter	After examination
Cases referred to vascular center	After examination

The web-based protocol was finalized in October 2020, and the first persons were invited to the screening facility in October 2020. The COVID-19 pandemic was unfortunately reaching its second surge, and the study was halted in between November 2020 and March 2021. Due to the pandemic, the study inclusion has been started and halted in three subsequent periods. During spring term 2022, the study would reach completion with 1500 ultrasound-examined persons.

## Discussion

The only risk group currently targeted with population-based screening is elderly men. With falling AAA rates among elderly men, subsequent to falling male smoking rates, it is likely that proportionally more AAAs will be found outside of this traditional risk group. The rationale for targeted screening in this form is that targeted screening will be expanded to another high-prevalence risk group and also include both men and women. Several aspects can be emphasized when discussing the possible importance by the DAAAD project, particularly if a health equality perspective is included. This not previously explored study design for high-risk vascular groups can be feasible and cost-effective. Such selective screening route for this high-risk group, including both women and men is not performed systematically in any other country despite guidelines suggesting targeted screening. Secondly, a presumed high-risk group is finally investigated for their true risk of disease [4, 16]. The sisters and brothers to AAA patients are already identified as a high-risk group, and a detection screening program is shown to be cost-efficient [15].

The plausible high risk for adult offspring of AAA patients to develop AAA has not been evaluated scientifically. The possibility to detect them by hospital-based nurse routes is presumably extremely difficult as compared to sibling screening, since the age-gap minimizes the chances to identify and screen them at a reasonable age (above 50 years).

This program will evaluate the risk for AAA in adult offspring and also evaluate a highly probable effective registry-based detection route. This could be more cost-efficient than any other AAA screening program, since the prevalence presumably is very high, and the registry-based route could be more cost-efficient than nurse-based detection.

The ultimate benefit of this program will be a crude reduction of sudden deaths from AAA among adult offspring to AAA patients, and this will be specifically impressive for the female relatives that are never subjected to any AAA-screening in Sweden.

### Limitations, challenges, and risks

The efficacy of a population based or targeted screening program depends on several major factors, modified after the WHO requirements for screening. First, the target population must accept and adhere in sufficient number; this can be interpreted as the participation rate. For other Swedish screening programs such as colorectal cancer, it is 68% and for cervix screening 60–70% [22, 23]. In the regional program for AAA in men, it is 75% [24]. Since this study was performed in the midst of a pandemic with formal restrictions, this will influence the participation rate which in turn will influence the transferability to a general population in a non-pandemic setting. Secondly, the cost of the program must be affordable considering the lives saved by the program. Here, the gold standards for Sweden are the NBHW recommended limits for screening [7]. Thirdly, if the participation is below 40%, the true prevalence in the target population can be discussed, but presumably, based on previous publications, this is lower in the participating cohort than among the invited non-participants. The study will then generate an underestimation of the benefit of a program. One must also evaluate if one introduces harm to the invited cohort due to a possible increase in anxiety for invited or diagnosed persons due to the information on the risk for this disease; this could be especially profound for persons with a personal history of a parents with a ruptured AAA. This would also be addressed and minimized by participating and thereby obtain a solid examination of the personal occurrence for AAA.

## Conclusion

In conclusion, this feasibility study will be instrumental in supporting the development of a possible new model to invite persons with high risk to develop hereditary rare diseases. To our knowledge, this is a unique, potentially safe, and probably a cheap model to invite targeted risk groups for selected screening. If the study design and the results are shown to be cost-effective at the detected participation rate and prevalence, it should be further evaluated and adopted to a national program. The model also invites women and men, which is unique for this specific patient group, considering that all population-based programs only include men.

## Supplementary Information

**Additional file 1: Supplemental Table 1.** Diagnostic codes for Extraction by codes for AAA. Identification by coding: 1987-2019 in ICD 9 and 10.

## Data Availability

The approval of the study is given with the assurance that all collected data are anonymized and untraceable. The data will not be shared publicly and will be analyzed on group-level.

## Abbreviations

AAA: Abdominal aortic aneurysm
DAAAD: Detecting AAA in First Degree Relatives (Adult Offspring) to AAA patients
EQ-5D-5L: EuroQol-5 Dimensions Scale (5 level), European
FDR: First-degree relative
ICD: International Classification of Diseases
NBHW: Swedish National Board of Health and Welfare
US: Ultrasound
