# Supplementary material for: Evaluating feasibility of using national registries for identification, invitation, and ultrasound examination of persons with hereditary risk for aneurysm disease—detecting abdominal aortic aneurysms in first degree relatives (adult offspring) to AAA patients (DAAAD)
Source: Pilot Feasibility Stud. 2022 Dec 12;8:252. doi: 10.1186/s40814-022-01196-9 (PMC9742022; doi:10.1186/s40814-022-01196-9)
Supplement: Supplementary file 1 — Additional file 1: Supplemental Table 1. Diagnostic codes for Extraction by codes for AAA. Identification by coding: 1987-2019 in ICD 9 and 10. [file 40814_2022_1196_MOESM1_ESM.docx]

**Supplemental**

**Supplemental Table 1. Diagnostic codes for Extraction by codes for AAA. Identification by coding: 1987-2019 in ICD 9 and 10.**

|  | ICD-10 |
| --- | --- |
| Diagnostic code:  and or: | I71.4 or I71.3 |
| Treatment code: | PDQ10, PDQ21, PDG10, PDG20, PDG21, PDA10, PDG22, PDG23, PDQ21, PDH10, PDH21 |
|  | **ICD- 9** |
| Diagnostic code  and or: | Aneurysma  440,00 Aortae  440,99 Universalis sive NUD Aortaaneurysm (non-syfilitic)  441,20 Abdominale, dissecanti non indicato  442,00 Cum ruptura  442,09 Ruptura non indicate |
| Treatment code: | 8807 elective AAA operation  0961 emergent operation suspected rupture  0962 emergent rupture operation |
